# MLVA16 Typing of Portuguese Human and Animal *Brucella melitensis* and *Brucella abortus* Isolates

**DOI:** 10.1371/journal.pone.0042514

**Published:** 2012-08-14

**Authors:** Ana Cristina Ferreira, Lélia Chambel, Tania Tenreiro, Regina Cardoso, Lídia Flor, Isabel Travassos Dias, Teresa Pacheco, Bruno Garin-Bastuji, Philippe Le Flèche, Gilles Vergnaud, Rogério Tenreiro, Maria Inácia Corrêa de Sá

**Affiliations:** 1 Laboratório Nacional de Investigação Veterinária, Instituto Nacional de Recursos Biológicos, I.P., Lisboa, Portugal; 2 Centro de Biodiversidade, Genómica Integrativa e Funcional, Universidade de Lisboa, Faculdade de Ciências, Lisboa, Portugal; 3 Laboratório Regional de Veterinária, Vinha Brava, Angra do Heroísmo, Azores, Portugal; 4 Laboratório de Microbiologia, Centro Hospitalar de Lisboa Ocidental, Lisboa, Portugal; 5 Agence National de Sécurité Sanitaire Alimentation Environnement Travail, Maisons-Alfort, France; 6 Université Paris-Sud, Institut de Génétique et Microbiologie, Orsay, France; 7 CNRS, Orsay, France; 8 DGA/Maitrise NRBC, Vert le Petit, France; 9 Mission pour la Recherche et l'Innovation Scientifique, Bagneux, France; Institut National de la Recherche Agronomique, France

## Abstract

To investigate the epidemiological relationship of isolates from different Portuguese geographical regions and to assess the diversity among isolates, the MLVA16_Orsay_ assay (panels 1, 2A and 2B) was performed with a collection of 126 *Brucella melitensis* (46 human and 80 animal isolates) and 157 *B. abortus* field isolates, seven vaccine strains and the representative reference strains of each species. The MLVA16_Orsay_ showed a similar high discriminatory power (HGDI 0.972 and 0.902) for both species but panel 1 and 2A markers displayed higher diversity (HGDI 0.693) in *B. abortus* compared to *B. melitensis* isolates (HGDI 0.342). The *B. melitensis* population belong to the “Americas” (17%) and “East Mediterranean” (83%) groups. No isolate belonged to the “West Mediterranean” group. Eighty-five percent of the human isolates (39 in 46) fit in the “East-Mediterranean” group where a single lineage known as MLVA11 genotype 116 is responsible for the vast majority of *Brucella* infections in humans. *B. abortus* isolates formed a consistent group with bv1 and bv3 isolates in different clusters. Four MLVA11 genotypes were observed for the first time in isolates from S. Jorge and Terceira islands from Azores. From the collection of isolates analysed in this study we conclude that MLVA16_Orsay_ provided a clear view of *Brucella* spp. population, confirming epidemiological linkage in outbreak investigations. In particular, it suggests recent and ongoing colonisation of Portugal with one *B. melitensis* lineage usually associated with East Mediterranean countries.

## Introduction

Brucellosis is a worldwide zoonotic disease caused by *Brucella*, a gram-negative alphaproteobacteria that infects a wide range of mammal including domestic and wild animals as well as humans [1]. Currently, there are ten recognized species of *Brucella* based on phenotypic and antigenic differences, differential host specificity and genetic diversity. The classical *Brucella* species were: *B. abortus* (cattle); *B. melitensis* (sheep and goats); *B. ovis* (sheep); *B. canis* (dogs); *B. suis* (pigs, wild boars, hares, reindeer and caribou) and *B. neotomae* (rodent) [2]. Recently, four additional species have been identified, namely *B. pinnipedialis* isolated from pinnipeds, *B. ceti* isolated from cetaceans [3], *B. microti* isolated from voles, fox and soil [4] and *B. inopinata* isolated from a woman' breast implant [5]. In three species biovars are recognized, *B. abortus* (1–6, 9), *B. melitensis* (1–3) and *B. suis* (1–5) [6].

Brucellosis in humans is largely dependent on the animal reservoir and can occur by direct contact with infected animals or through consumption of contaminated dairy products. *B. melitensis* and *B. abortus* are highly pathogenic and a frequent cause of human infection. *B. abortus* is the most disseminated species worldwide and *B. melitensis* produces the most severe infection in humans and predominates in the Mediterranean Basin [7].

Although several countries have implemented eradication programs which resulted in the reduction or elimination of the disease, the infection remains enzootic in many regions of the world [8]. In the scope of eradication programmes *Brucella* species and biovars are currently typed by microbiological, serological and chemical tests. For many years, the phenotypic characterization was one of the few tools available to provide epidemiological data but does not allow tracing back the source of origin, hindering the application of measures to prevent additional spread and disease elimination from the primary source [9], [10]. In the countries where brucellosis has been eradicated or strictly controlled, continued surveillance is essential to prevent the re-emergence of the disease [8].

Genetic typing of the bacterium is often used for molecular epidemiological purposes and can be useful to observe population structures. In the last decades, attempts made to develop epidemiological markers for *Brucella* have shown limited success. Most of the tests lacked reproducibility and showed a moderate capability to differentiate strains [9], [11]. The genetic conservation within *Brucella* has resulted in past difficulties in establishing the true relationships between some classical *Brucella* species and biovars and in identifying molecular markers for certain groups. In recent years, the sequencing of multiple genetic loci (Multilocus Sequence Typing; MLST) has rapidly gained acceptance as a tool for the characterization of microbial populations. MLST has provided the first genus-wide and robust view of the population structure of this highly clonal genus [10]. Over the past few years, polymorphic tandem repeat loci have been identified by the analysis of published genome sequences of the strains *B. melitensis* biovar 1 16 M, *B. suis* biovar 1 1330 and *B. abortus* biovar 1 9–941 [12]–[17]. One of the applications based on these markers is the multiple-locus variable number tandem repeats analysis (MLVA) with a set of 16 repeat loci (MLVA16_Orsay_) [9], [13]. MLVA has a much higher discriminatory power than MLST and produces highly relevant clustering. A number of reference strains have been typed by both MLVA and MLST [6]. These linking strains make it possible to predict with high confidence a MLST type from the MLVA type for most new isolates. Only those strains showing weak clustering to such a linking strain will need to be analysed by the more demanding MLST or draft whole genome sequencing analysis to become new linking strains.

In Portugal, brucellosis is an endemic disease where *B. melitensis* biovars 1 and 3 and *B. abortus* biovars 1 and 3 are the prevailing species (LNIV, National Laboratory for Veterinary Research, NRL for Brucellosis, unpublished data). Human infections due to *B. melitensis* pose a significant public health problem with 80 confirmed cases in 2009 (Portuguese Health Authority, unpublished data). The archipelago of Azores is officially free of *B. melitensis* but *B. abortus* is present in three of the nine islands, S. Miguel, S. Jorge and Terceira. Animals move between the archipelago islands and from the islands to mainland Portugal [18]. In this report we present the results of a study performed by *Brucella* MLVA16_Orsay_ on 283 human and animal field isolates of *B. melitensis* and *B. abortus*. The aims of this study were to evaluate MLVA16_Orsay_ and its MLVA11 subset in order to assess the diversity among strains for epidemiological purposes in human brucellosis and to estimate the epidemiological relationship of isolates from different geographical origins in Portugal.

## Results

### MLVA 16 typing and clustering of *B. melitensis* and *B. abortus* populations

**Figure 1:**
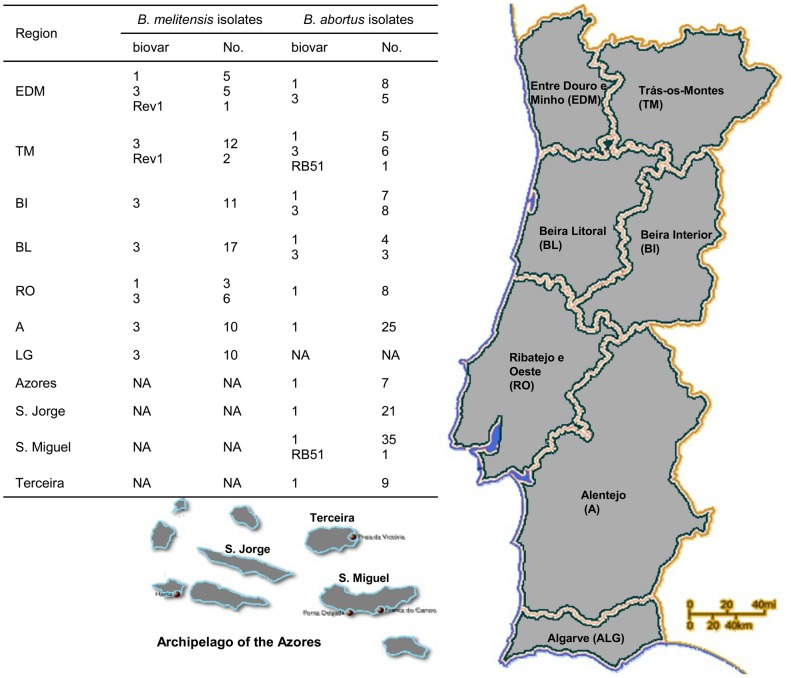
Distribution of animal *B. melitensis* and *B. abortus* isolates among Portugal regions.

The MLVA16_Orsay_ assay (panels 1, 2A and 2B) was performed with a collection of 126 *B. melitensis* (46 human and 80 animals) and 157 *B. abortus* isolates, 7 vaccine strains and the representative reference strains of each species (Table 1, Figure 1). All loci could be amplified in all samples, except for three *B. abortus* isolates, one negative for bruce09, another for bruce16 and a third for bruce30. The combination of the three panels (1, 2A and 2B) provided a similar high discriminatory power in the *B. melitensis* and *B. abortus* populations (HGDI of 0.972 and 0.902, respectively) (Table 2) with a genetic similarity coefficient ranging from 69.3 to 100% for *B. melitensis* and 79.3 to 100% for *B. abortus*. The corresponding HGDI for the MLVA11 subset of loci (Panels 1 and 2A) were 0.342 and 0.693 for *B. melitensis* and *B. abortus* respectively (Table 2).

**Table 1:** *Brucella* strains used in this work.

Biovar	No. of isolates	Origin	Source
***Brucella melitensis*** ** field strains**			
1	7	Portugal (4 isolated in France)	Human
	8	EDM, RO	3 Cattle; 1 Goat; 4 Sheep
3	38	Portugal (18 isolated in France)	Human
	72	A, ALG, BI, BL, EDM, RO, TM	20 Cattle; 20 Goat; 46 Sheep
Rough	1	Portugal (isolated in France)	Human
***Brucella abortus*** ** field strains**			
1	129	Azores, A, BI, BL, EDM, RO, TM	Bovine
3	28	A, BI, EDM, TM	Bovine
***Brucella*** ** vaccine strains**			
*B. melitensis* Rev1	3	EDM, TM	1 Goat; 2 Sheep
*B. abortus* RB51	2	Azores, TM	Bovine
*B. abortus* RB51	1	Commercial	NA
*B. abortus* S19	1	Commercial	NA
***Brucella*** ** reference strains**			
**Strain**	**Biovar**	**Reference**	**Host**
*B. melitensis* 16 M	1	ATCC 23456	Goat
*B. melitensis* 63/9	2	ATCC 23457	Goat
*B. melitensis* Ether	3	ATCC 23458	Goat
*B. abortus* 544	1	ATCC 23448	Cattle
*B. abortus* 86/8/59	2	ATCC 23449	Cattle
*B. abortus* Tulya	3	ATCC 23450	Human
*B. abortus* 292	4	ATCC 23451	Cattle
*B. abortus* B3196	5	ATCC 23452	Cattle
*B. abortus* 870	6	ATCC 23453	Cattle
*B. abortus* C68	9	ATCC 23455	Cattle
*B. abortus* 2308	1	Dr. J.M. Blasco; CITA	Cattle

A, Alentejo; ALG, Algarve; BI, Beira Interior; BL, Beira Litoral; EDM, Entre Douro e Minho; RO, Ribatejo e Oeste; TM, Trás-os-Montes; ATCC, American Type Culture Collection; CITA, Centro de Investigación y Tecnología Agro Alimentaria del Gobierno de Aragón; NA, Not Applicable.

**Table 2:** Hunter-Gaston Diversity Index (HGDI) for the 288 *Brucella* isolates (excluding vaccine strains).

	126 *B. melitensis*			157 *B. abortus*		
Locus	No. of alleles	HGDI^b^	CI 95%^c^	No. of alleles	HGDI^b^	CI 95%^c^
MLVA-16	61	0.972	0.959–0.985	39	0.902	0.871–0.934
MLVA-11	13	0.342	0.235–0.450	7	0.693	0.645–0.740
Panel1	7	0.314	0.210–0.417	6	0.645	0.592–0.697
bruce06	2 (1,3)	0.291	0.204–0.377	2 (3,4)	0.109	0.044–0.173
bruce08	2 (4,5)	0.291	0.204–0.377	1 (5)	0.000	0.000–0.045
bruce11	1 (3)	0.000	0.000–0.056	2 (3,4)	0.295	0.218–0.372
bruce12	3 (11,13,14)	0.107	0.034–0.179	2 (12,13)	0.013	0.000–0.037
bruce42	4 (2–5)	0.307	0.208–0.406	2 (1,2)	0.422	0.365–0.480
bruce43	1 (2)	0.000	0.000–0.056	1 (2)	0.000	0.000–0.045
bruce45	1 (3)	0.000	0.000–0.056	1 (3)	0.000	0.000–0.045
bruce55	2 (2,3)	0.291	0.204–0.377	2 (1,3)	0.295	0.218–0.372
Panel 2A	8	0.326	0.230–0.423	2	0.097	0.036–0.159
bruce18	4 (4–7)	0.309	0.217–0.400	1 (6)	0.000	0.000–0.045
bruce19	3 (18,20,22)	0.293	0.205–0.382	2 (20,21)	0.097	0.036–0.159
bruce21	3 (5,6,8)	0.293	0.205–0.382	1 (8)	0.000	0.000–0.045
Panel 2B	55	0.970	0.957–0.982	32	0.832	0.780–0.884
bruce04-TR6^a^	7 (2–7,9)	0.759	0.712–0.807	3 (3–5)	0.448	0.377–0.518
bruce07	7 (4–10)	0.743	0.691–0.796	4 (4–7)	0.391	0.305–0.478
bruce09-TR8^a^	5 (3,5–8)	0.309	0.209–0.409	7 (0,3,5–8,12)	0.313	0.224–0.402
bruce16	6 (3–8)	0.773	0.726–0.820	6 (0,3–6,8)	0.557	0.481–0.633
bruce30-TR2^a^	5 (4–8)	0.401	0.301–0.502	6 (0,3–7)	0.631	0.567–0.694

a Markers from HOOF-print analysis described by Bricker *et al.*, 2003.

b Hunter and Gaston index.

c Precision of the diversity index, expressed as 95% upper and lower boundaries.

**Figure 2:**
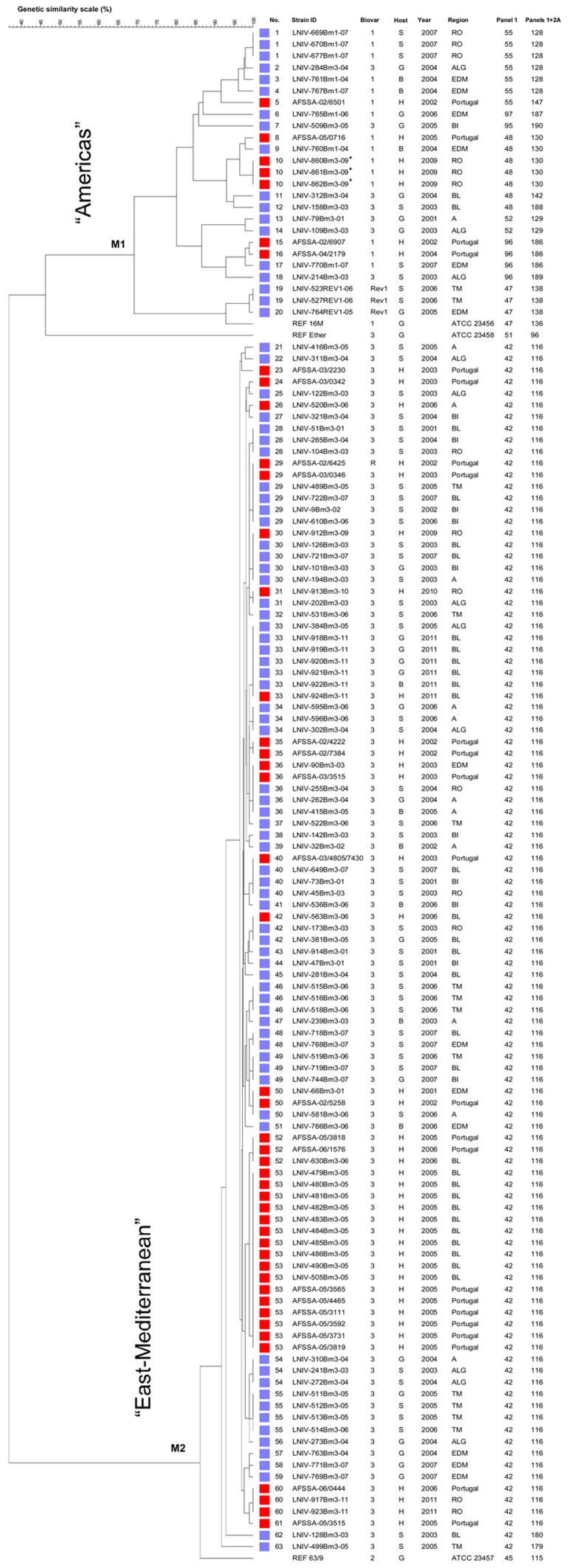
Cluster analysis for 132 *B. melitensis* strains based on the data set of MLVA16_Orsay_. In the columns, the following data for isolates are indicated: genotype numbering (No.), strain identification (ID), biovar (Bv.), host (B, bovine; G, goat; H, human; S, sheep), year of isolation (Year), geographic region (Region), Panel1 and MLVA11 (Panels1+2A) genotypes corresponding to each isolate in the Brucella2010 database for each set of loci. A total of 63 genotypes were observed. The red colour is referring to human isolates and the blue colour to animal isolates. **A**, Alentejo; **ALG**, Algarve; **BI**, Beira Interior; **BL**, Beira Litoral; **EDM**, Entre Douro e Minho; **RO**, Ribatejo e Oeste; **TM**, Trás-os-Montes. Isolates recovered from the same patient are marked with an asterisk.

The 126 *B. melitensis* field strains were distributed into 13 MLVA11 genotypes and 61 MLVA16_Orsay_ genotypes. Seven MLVA11 genotypes, numbered 179, 180 and 186 to 190, not previously reported, represented only 9 isolates (genotype 186 occurred in two human and one sheep isolates and genotypes 179, 180, 187–190 in single animal isolates). Two clearly distinct clusters could be defined, M1 and M2 (Figure 2). Cluster M1 covered 20 MLVA16_Orsay_ genotypes comprising 22 field isolates (genotypes 1 to 18), three Rev1 isolates (genotypes 19–20) and the 16 M reference strain, corresponding to eleven MLVA11 genotypes (128, 147, 187, 190, 130, 142, 188, 129, 186, 189 and 138). Cluster M2 contained the reference strain 63/9 and 104 isolates grouped in 42 MLVA16_Orsay_ genotypes, numbered 21 to 63. All M2 isolates shared the MLVA11 genotype 116, with only two exceptions, corresponding to the new genotypes, 179 and 180. These genotypes were single locus variants of MLVA11 genotype 116. Cluster M1 was more diverse as illustrated on Figure 2.

**Figure 3:**
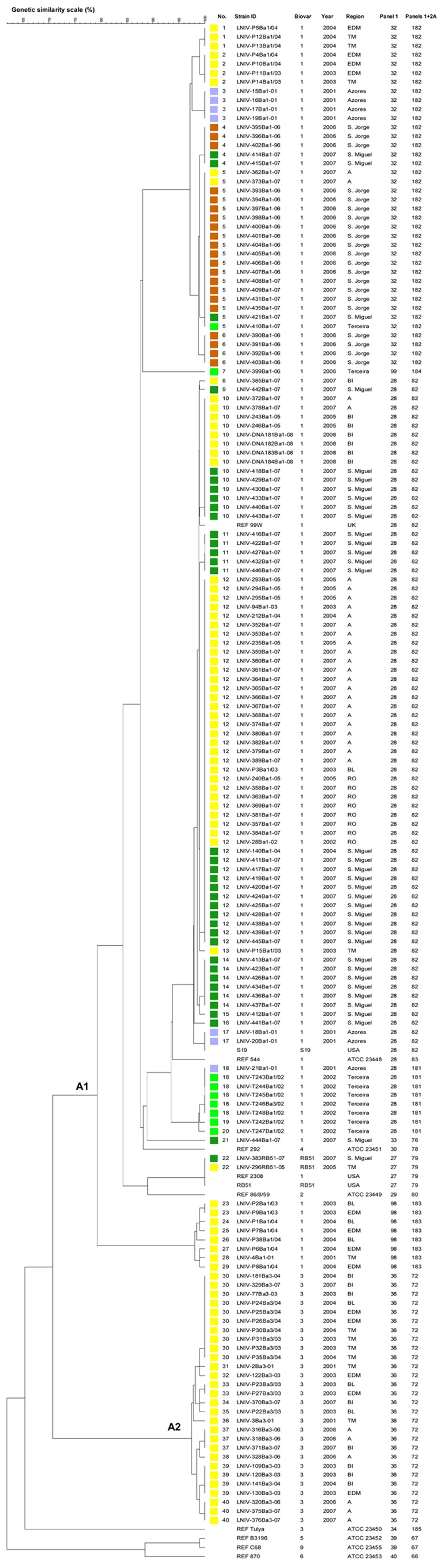
Minimum Spanning Tree analysis of published *B. melitensis* isolates (excluding vaccine strains) using the MLVA11 data. Colour codes are associated with the main *B. melitensis* MLVA groups. The analysis was performed using all isolates from this study and typing data from more than one thousand isolates previously published [19]–[22], Brucella2010 database hosted at http://mlva.u-psud.fr]. Portuguese isolates are shown in yellow. The *B. melitensis* population is included in “American” (17%) and “East-Mediterranean” (83%) groups. Thirty nine human isolates (85%; 39 out of 46) are MLVA11 genotype 116 usually associated with the “East-Mediterranean” *B. melitensis* strains and seven isolates (15%) are included in the “American” group. The numbers represent the MLVA11 genotypes found in this study. The size of the shapes indicates the number of strains described in each genotype. Each of the circles showing yellow and dark blue or red colors included the Portuguese genotype (yellow) and the genotypes found in “East-Mediterranean” (dark blue) or “American” (red) *B. melitensis* isolates.

One hundred fifty seven bovine *B. abortus* field strains were studied. MLVA11 discriminated seven genotypes (not including the reference and vaccine strains), four of which were not previously observed (181 to 184). MLVA16_Orsay_ distinguished a total of 40 genotypes with 20 singleton genotypes (Figure 3). The isolates fell into two clusters, A1 and A2 respectively. Cluster A1 corresponded to 29 MLVA16_Orsay_ genotypes (1 to 29 containing the bv1 (544, 2308 and 99 W), bv2 (86/8/59) and bv4 (292) strains, the vaccine strains S19 and RB51 and all *B. abortus* bv1 field isolates. The vast majority of A1 isolates belonged to MLVA11 genotype 82, 181, 182 or 183. MLVA11 genotype 82 was observed only in mainland Portugal and S. Miguel island. MLVA11 genotype 181 seems to be specific to Terceira. MLVA11 genotype 182 was observed in mainland Portugal, S. Miguel, Terceira and S. Jorge islands. Cluster A2 grouped all isolates from bv3 (genotypes 30 to 40). These isolates were from mainland and shared MLVA11 genotype 72. *B. abortus* bv1 and bv3 isolates were clearly differentiated by bruce11 (4 and 3 repeats respectively) and bruce55 (3 and 1 repeats respectively).

### Epidemiological relationship between human and animal *B. melitensis* isolates

Forty six *B. melitensis* strains isolated from Portuguese patients and 80 *B. melitensis* strains from cattle, sheep and goats from the different Portuguese regions (Figure 1) were studied. The human isolates represented 21 MLVA16_Orsay_ genotypes, thirteen of which included only human isolates and eight were common to human and animals (Figure 2). Seven human isolates belonged to cluster M1 and 39 to M2. The M1 isolates were associated with three MLVA11 genotypes, 130, 147, 186. All the other 39 human isolates showed MLVA11 genotype 116. Cluster analysis grouped three isolates, with MLVA16_Orsay_ genotype 10, obtained from three different cultures from one patient (strains LNIV-860Bm3-09, LNIV-861Bm3-09 and LNIV-862Bm3-09). In some cases an association with isolation year and geographic origins was observed. Sixteen human isolates in cluster M2 shared the genotype 53 (10 isolates were from an outbreak in Beira Litoral (BL) in 2005 and the other six were isolated in France from Portuguese emigrants, in the same year). Additionally, three human strains (one from BL and two isolated in France) clustered as genotype 52, which differs from genotype 53 by the loss of two repeat units at locus bruce16. Six strains (one human, four goat and one bovine isolates) from a recent outbreak (2011) in BL, as well as a sheep strain isolated in 2005 in Algarve (ALG) displayed the genotype 33. The genotype 60 clustered a human strain isolated in France in 2006 and the human strains LNIV-917Bm3-11 and LNIV-923Bm3-11, isolated from the same patient before and three months after treatment with rifampicin and doxycycline.

**Figure 4:**
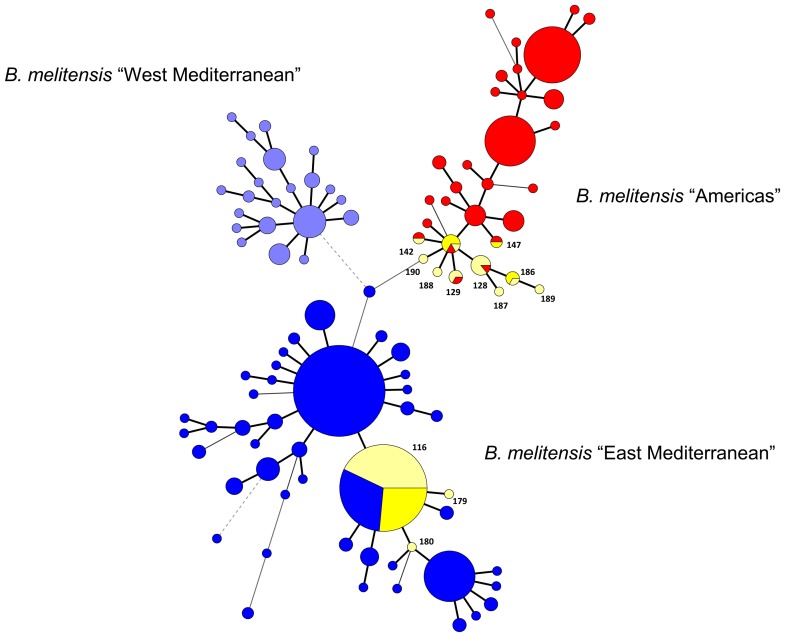
Cluster analysis for 170 *B. abortus* strains based on the data set of MLVA16_Orsay_. In the columns, the following data for isolates are indicated: genotype numbering (No.), strain identification (ID), biovar (Bv.), year of isolation (Year), geographic region (Region), Panel1 and MLVA11 (Panels1+2A) genotypes corresponding to each isolate in the Brucella2010 database for each set of loci. The colour code reflects the Portuguese regions: Portugal mainland, yellow; Azores, light blue; S. Miguel, dark green; S. Jorge, orange; Terceira, green.A total of 40 genotypes were observed. **A**, Alentejo; **BI**, Beira Interior; **BL**, Beira Litoral; **EDM**, Entre Douro e Minho; **RO**, Ribatejo e Oeste; **TM**, Trás-os-Montes.

A minimum spanning tree (MST) analysis was performed using all isolates from this study and typing data from more than one thousand isolates previously published in Brucella2010 database hosted at http://mlva.u-psud.fr and by other authors [19]–[22]. Seven human (15%) and 15 animal (19%) isolates were included in the “Americas” group. Most of the Portuguese strains clustered in the “East Mediterranean” group with 39 human (85%) and 63 animals (79%) isolates sharing the genotype 116 (Figure 4). None belonged to the “West Mediterranean” group.

### Epidemiological relationship between *B. abortus* isolates from different geographical origins

A total of 157 bovine *B. abortus* isolates from the seven Portuguese mainland regions (Figure 1) and S. Miguel, S. Jorge and Terceira islands from Azores were evaluated based on MLVA16_Orsay_ data (Figure 4). Isolates from Ribatejo e Oeste (RO), as well as most of the isolates from Alentejo (A) clustered in genotype 12, irrespective of the isolation year. Further, in Beira Interior (BI) two strains (LNIV-243Ba1-05 and LNIV-246Ba1-05) from an outbreak in 2005 and four strains (LNIV-DNA181Ba1-08, LNIV-DNA182Ba1-08, LNIV-DNA183Ba1-08 and LNIV- DNA184Ba1-08), isolated from a neighbouring herd in 2008 displayed the genotype 10. Strains from Azores clustered in 18 genotypes. S. Miguel isolates were grouped in 11 genotypes which were also found in isolates from mainland (Figure 3) and S. Jorge isolates clustered apart from most of the other Azores and mainland strains (genotypes 4, 5 and 6). One strain from Terceira (LNIV-399Ba1-06, genotype 7) showed 13 repeats in bruce12, one more than the other isolates. Eight milk isolates, also from Terceira, presented 20 repeats in bruce19 (genotypes 18, 19 and 20), one less than the other strains isolated from tissues. Additionally, two isolates of RB51 (1 from S. Miguel and 1 from Trás-os-Montes) displayed the genotype 22, like the RB51 vaccine strain and its parental strain 2308. These strains had three unit repeats in bruce43, one more compared to all the other bv1 isolates.

**Figure 5:**
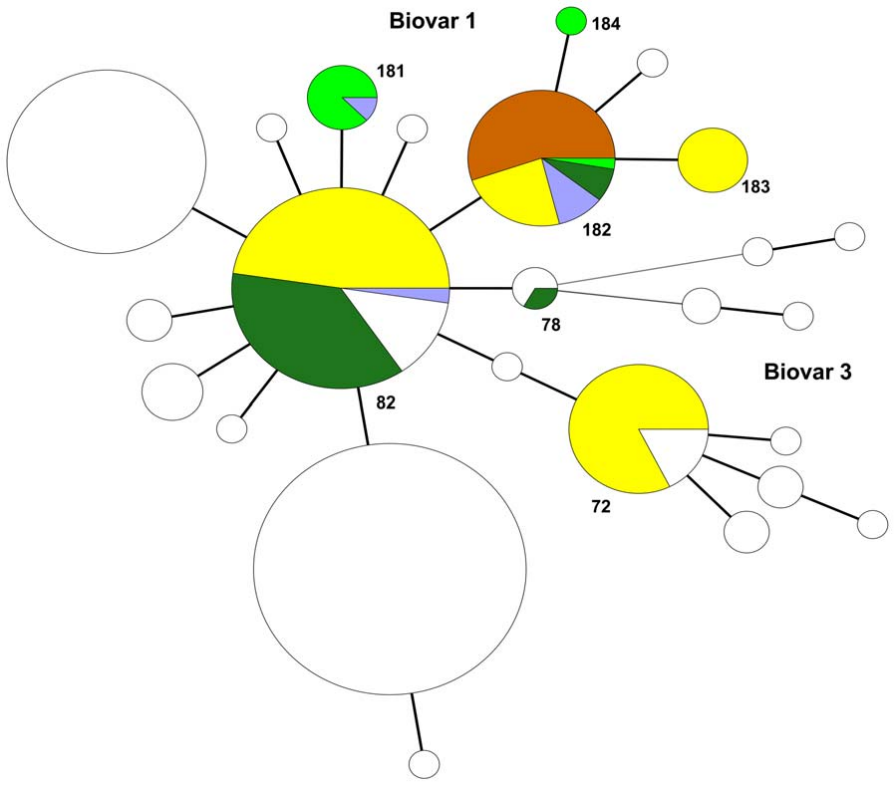
Minimum Spanning Tree analysis of published *B. abortus* isolates using the MLVA11 data. The data were recovered from the published *Brucella*2010 MLVA database. Colour codes are associated with Portuguese *B. abortus* genotypes: Portugal mainland, yellow; Azores, light blue; S. Miguel, dark green; S. Jorge, orange; Terceira, green. Circles in white represent genotypes from other countries and previously published in Brucella2010 MLVA database. The numbers represent the MLVA11genotypes found in this study. The size of the shapes indicates the number of strains described in each genotype. Each of the circles showing more than one colour includes the Portuguese genotypes and others previously published.

A MST analysis using all *B. abortus* isolates was performed with previously published typing data recovered from the compilation in Brucella2010 database (Figure 5).

## Discussion

Control of brucellosis, particularly in the final stages of an eradication program, requires an accurate surveillance and highly discriminatory methods to characterize an outbreak strain and determine the infection source and transmission routes. Correct application of bacterial typing will increase the efficacy of control measures aimed to contain or interrupt the outbreak. For epidemiological trace-back purposes, the array of phenotypic tests used in the classification of *Brucella* species and biovars is not accurate enough particularly in geographical regions with a predominance of a small number of biovars, like Portugal, where most isolates are *B. abortus* bv1 and bv3 and *B. melitensis* bv1 and bv3.

MLVA16_Orsay_ and its MLVA11 subset were used to assess the diversity among strains for epidemiological purposes in human brucellosis and to estimate the epidemiological relationship between *B. melitensis* (n = 126) and *B. abortus* (n = 157) isolates collected during the last 11 years from different geographical areas in Portugal. The combination of the three panels provided a similar high discriminatory power for both species (HGDI 0.972 and 0.902). However, considering panel 1 and 2A markers (MLVA11), *B. abortus* population displayed a higher diversity (HGDI 0.693) than *B. melitensis* (HGDI 0.342). The reduced diversity of *B. melitensis* isolates in Portugal is surely related with the homogenous population where a single lineage, MLVA11 genotype 116, is spreading.

Comparing *B. melitensis* MLVA11 results with typing data published by others [19]–[22] and with Brucella2010 database, seven genotypes (179, 180, 186–190) were recorded for the first time. No isolates belonged to the “West Mediterranean” group. Eighty three percent of isolates were included in the “East Mediterranean” and 7% in the “Americas” group. All Portuguese isolates (human and animal) included in the “East Mediterranean” group shared panel 1 genotype 42 which is also widely distributed throughout Spain and predominant in Turkey [9], [20], [22]. Eighty-five percent of human isolates of this group were assigned to the MLVA11 genotype 116, but when comparing with other Mediterranean countries, only 29% of Spanish, 28.5% of French and 16% of Turkish strains clustered in this genotype. The genetic homogeneity of the Portuguese *B. melitensis* population, unlike other Mediterranean countries, suggests a recent evolution from a common ancestor in Portugal. The “Americas” group shows high diversity. It is tempting to speculate that this may have been the predominant lineage in Portugal at the time of the colonization of Southern America. This historical lineage may currently be undergoing replacement by a new lineage represented by MLVA11 genotype 116 presumably recently imported to Portugal.

The cluster analysis of *B.melitensis* isolates identified two major clusters (M1 and M2). This species forms a heterogeneous group that fits moderately with the biotyping results. Considering MLVA11, *B. melitensis* bv3 isolates are separated from bv3 reference strain (Ether), differing from bv2 reference strain (63/9) in one locus (bruce12). Similar results were obtained by other authors and methods [1], [23], questioning the phylogenetic robustness of some biovars established by classical microbiological typing.

MLVA16_Orsay_ was able to identify the epidemiological relationship between strains. Genotype 53, assigned to 16 human isolates (10 from an outbreak occurred in 2005 in Beira Litoral and six isolated from Portuguese emigrants, in the same year, in France) probably represents an outbreak. Additionally, three human isolates (one from Beira Litoral and two from France, both isolated in 2005) clustered together as genotype 52, which differs from genotype 53 by the loss of two repeat units at the highly variable locus bruce16, suggesting a close relatedness [24], [25]. The six strains from the recent outbreak (2011) in Beira Litoral (one human, four goats and one bovine isolate) share genotype 33, clearly showing the epidemiological relation between isolates from human and animal origin. A sheep strain isolated in 2005 in Algarve displayed the same genotype 33. These strains had the same MLVA16_Orsay_ profile as a strain isolated in France in 1996 (presumably from a Portuguese patient) and described by Al Dahouk *et al*. [9], suggesting a common source of infection and confirming the persistence of genotypes in certain areas for long periods. A previous isolate (LNIV- 917Bm3-11) and the strain LNIV-923Bm3-11, recovered from the same patient after antibiotic treatment presented genotype 60. These results confirm the relapse even after three months of treatment with rifampicin and doxycycline. The antibiotic minimal inhibitory concentration (MIC) was determined by agar dilution method, as recommended by the Clinical Laboratory Standards Institute [26] and both strains exhibited the same susceptibility to rifampicin and doxycycline (MIC<1 mg/mL).

*B. abortus* strains formed a consistent group with bv1 and bv3 isolates completely separated in clusters A1 and A2. As noticed by other authors [27], *B. abortus* bv3 isolates are found in a separate group from the biovar reference strain (Tulya). When comparing MLVA11 results with typing data published in Brucella2010 database, seven genotypes were defined. Genotypes 181 to 184 were observed for the first time in isolates from S. Jorge and Terceira islands. All *B. abortus* bv3 isolates presented the same MLVA11 genotype 72 previously described by Le Flèche *et al.* [13].

The epidemiological relationship between isolates from different Portuguese geographical origins was evaluated by MLVA16_Orsay_. S. Jorge isolates clustered in three genotypes (4, 5 and 6), separately from most of the other strains from Azores and mainland, which differ from each other only in one repeat in bruce16 or in bruce30. The one repeat difference in the highly variable microsatellites from panel 2B reveals them as the same strain, presumably with a recent common ancestor. These genotypes were also found in two isolates from S. Miguel, one from Terceira and two from Alentejo showing the importance to monitor national movement of strains by MLVA16_Orsay_. Further, Bruce42 distinguished these isolates from others from Azores and mainland. Seventeen out of 34 *B. abortus* isolates from S. Miguel clustered in two genotypes (12 and 14) along with strains from Alentejo, Ribatejo e Oeste and Beira Interior. These strains showing identical genotypic traits are clones with a common origin, revealing linkage of genotypes with the geographic origin [25]. These results also confirm the report by Batista and Nunes [18] indicating that there is a high cattle movement out of S. Miguel to mainland. In Beira Interior, two strains from an outbreak in 2005 and four strains isolated from a neighbouring herd in 2008 displayed the same genotype 10, again confirming the epidemiological linkage identified in outbreak investigations.

Considering the vaccine strains, three Rev1 field isolates from two sheep and one goat (genotypes 19 and 20) shared panel 1 genotype 47 described by García-Yoldi *et al*. [28] and differed from strain 16 M in bruce07, bruce09 and bruce18. Also two isolates from Azores without island identification, isolated in 2001, presented the same genotype as the vaccine strain S19 (genotype 17), which is not unexpected, since vaccination with S19 was used in Azores (S. Miguel and Terceira) until 1984 [29]. Two RB51 field isolates (one from S. Miguel and one from Trás-os-Montes) displayed the same genotype as the RB51 vaccine strain and its parental strain 2308 (genotype 22), which is in accordance with previous observations [13], [27]. These results confirm the genetic stability of Rev1, S19 and RB51 vaccine strains [13], [28]. Also, the RB51 strains differed from all other *B. abortus* isolates in bruce43.

The set of 16 loci used in MLVA16_Orsay_ showed to be extremely discriminant and highly efficient in distinguishing strains within a local outbreak. Though able to correctly differentiate regional geographically related *B. abortus* isolates from Azores, it could not distinguish *B. melitensis* strains from closely related geographical regions from mainland. In fact, data on animal movement in Portugal show that all mainland regions are connected.

MLVA16_Orsay_ provides a clear view of the *Brucella* population, confirming epidemiological linkage in outbreak investigations. In areas with low prevalence of brucellosis, the epidemiological answers given by MLVA16_Orsay_ are very important to prevent the expansion and achieve the eradication of the disease. In particular, our results suggest a recent and ongoing colonisation of Portugal with one lineage (MLVA11 genotype 116) usually associated with East Mediterranean countries. Draft whole genome sequencing of selected isolates may help decipher sub lineages within Portugal lineage 116 and provide estimates to date this contamination event.

The data produced in this work can be queried in the *Brucella* MLVA database release (starting from the Brucella2012 release at http://mlva.u-psud.fr).

## Materials and Methods

### *Brucella* isolates

Two hundred and eighty-three new isolates were investigated: 126 *B. melitensis* and 157 *B. abortus* isolates. Fifteen reference strains were included as controls: the type strains of the three biovars of *B. melitensis* (biovars 1, 2 and 3) and biovars 1–6 and 9 of *B. abortus* reference strains; *B. abortus* biovar 1 strains 2308 and 99 W; S19 and RB51 vaccine strains as well as three Rev1 and two RB51 vaccine field isolates. All isolates and strains used in this study are summarized in Table 1 and the geographical origin of isolates is shown in Figure 1. All isolates were biotyped according to standard bacteriological procedures [30] by the Bacteriological Service of Laboratório Nacional de Investigação Veterinária (INRB, I.P./LNIV, Portugal). Twenty-three human *B. melitensis* isolates from Portuguese patients were provided by the EU Reference Laboratory for Brucellosis (ANSES, Maisons-Alfort, France).

### *Brucella* MLVA16_Orsay_ assay. (i) DNA sample preparation

*Brucella* DNA was prepared using the High Pure PCR Template Preparation Kit (Roche Diagnostics, Germany); **(ii) PCR amplification**. PCR amplification was performed in a total volume of 15 µl containing 3 ng of DNA, 1× PCR Reaction Buffer, 1 U of Taq DNA polymerase (Roche Diagnostics, Germany), 200 µM of each dNTPs and 0.3 µM of each flanking primers, as described by Maquart *et al.* [6]. Amplifications were performed in a MyCycler thermal Cycler (Biorad, France). An initial denaturation step at 96°C for 5 min was followed by 30 cycles of denaturation at 96°C for 30 s, primer annealing at 60°C for 30 s and elongation at 70°C for 1 min. The final extension step was performed at 70°C for 5 min. Five microliter of amplification products were loaded on a 3% standard agarose gel to analyse panel 2A and 2B loci (tandem repeats with a unit length shorter than 8 bp) and on a 2% standard agarose gel for panel 1 loci (tandem repeats with a unit length larger than 10 bp) and run under a voltage of 8 V/cm for 120 min. Depending on the tandem repeat unit length, a 20 bp (20 bp PCR Molecular Ruler, Biorad, France) or 100 bp ladder was used as molecular size markers (100 bp DNA ladder, Invitrogen).

### Data analysis

The 16 loci have been classified in three panels, named panel 1, composed of 8 minisatellite loci (bruce06, bruce08, bruce11, bruce12, bruce42, bruce43, bruce45 and bruce55), panel 2A (bruce18, bruce19 and bruce21) and panel 2B (bruce04, bruce07, bruce09, bruce16 and bruce30) composed of three and five microsatellite markers, respectively. The total number of repeat units at each locus was determined by the correlation with the amplicon size according to previously published tables (Le Flèche *et al*. 2006 version 3.3: http://mlva.u-psud.fr *Brucella* support web site for MLVA typing). The copy number for each locus and each panel was managed as a character dataset using BioNumerics version 6.6 (Applied Maths, Belgium). Cluster analysis was based on the categorical coefficient and unweighted pair group method using arithmetic averages (UPGMA). Three different character data sets were defined (panel 1, 2A and 2B markers) as combined sets using the composite data set tool provided by BioNumerics. Different weightings were given to each panel according to Al Dahouk *et al.* [9]: panel 1 markers got an individual weight of 2 (total weight 16), panel 2A markers got an individual weight of 1 (total weight 3) and markers of panel 2B got an individual weight of 0.2 (total weight 1). The genetic diversity of each locus and respective panels was determined using the Hunter & Gaston diversity index (HGDI) [31] via the online tool V-DICE available at the HPA website (http://www.hpa-bioinformatics.org.uk/cgi-bin/DICI/DICI.pl).

## References

[1] Lamontagne J, Béland M, Forest A, Côté-Martin A, Nassif N, et al (2010) Proteomics-based confirmation of protein expression and correction of annotation errors in the *Brucella abortus* genome. BMC Genomics 11: 300. 20462421 10.1186/1471-2164-11-300 PMC2877026

[2] OIE, World Organization for Animal Health (2009) World Animal Health Information Database (WAHID) Interface. http://www.oie.int/wahis/public.php?page=home.

[3] Foster G, Osterman BS, Godfroid J, Jacques I, Cloeckaert A (2007) *Brucella ceti* sp. nov. and *Brucella pinnipedialis* sp. nov. for *Brucella* strains with cetaceans and seals as their preferred hosts. Int J Syst Evol Microbiol 57: 2688–2693. 17978241 10.1099/ijs.0.65269-0

[4] Scholz HC, Hubalek Z, Sedlácek I, Vergnaud G, Tomaso H, et al (2008) *Brucella microti* sp. nov., isolated from the common vole *Microtus arvalis* . Int J Syst Evol Microbiol 58: 375–382. 18218934 10.1099/ijs.0.65356-0

[5] Scholz HC, Nöckler K, Göllner C, Bahn P, Vergnaud G, et al (2010) *Brucella inopinata* sp. nov., isolated from a breast implant infection. Int J Syst Evol Microbiol 60: 801–8. 19661515 10.1099/ijs.0.011148-0

[6] Maquart M, Le Flèche P, Foster G, Tryland M, Ramisse F, et al (2009) MLVA16 typing of 295 marine mammal *Brucella* isolates from different animal and geographic origins identifies 7 major groups within *Brucella ceti* and *Brucella pinnipedialis* . BMC Microbiol 9: 145. 19619320 10.1186/1471-2180-9-145 PMC2719651

[7] Pappas G, Papadimitriou P, Akritidis N, Christou L, Tsianos EV (2006) The new global map of human brucellosis. Lancet Infect Dis 6: 91–99. 16439329 10.1016/S1473-3099(06)70382-6

[8] Bricker BJ, Ewalt DR, Halling SM (2003) *Brucella* ‘HOOF-Prints’: strain typing by multi-locus analysis of variable number tandem repeats (VNTRs). BMC Microbiol 3: 15. 12857351 10.1186/1471-2180-3-15 PMC183870

[9] Al Dahouk S, Flèche PL, Nöckler K, Jacques I, Grayon M, et al (2007) Evaluation of MLVA typing for human brucellosis. J Microbiol Methods 69: 137–145. 17261338 10.1016/j.mimet.2006.12.015

[10] Whatmore A (2009) Current understanding of the genetic diversity of *Brucella*, an expanding genus of zoonotic pathogens. Infectious, Genetics and Evolution 9: 1168–1184. 10.1016/j.meegid.2009.07.001 19628055

[11] Bricker BJ (2004) Molecular diagnostics of animal brucellosis: a review of PCR-based assays and approaches, p.25–51. In I. López-Goñi and I. Moriyón (ed.), *Brucella* molecular and cellular biology. Universidad de Navarra, Pamplona, Spain.

[12] Le Flèche P, Hauck Y, Onteniente L, Prieur A, Denoeud F, et al (2001) A tandem repeats database for bacterial genomes: application to the genotyping of *Yersinia pestis* and *Bacillus anthracis* . BMC Microbiol 1: 2. 11299044 10.1186/1471-2180-1-2 PMC31411

[13] Le Flèche P, Jacques I, Grayon M, Al Dahouk S, Bouchon P, et al (2006) Evaluation and selection of tandem repeat loci for a *Brucella* MLVA typing assay. BMC Microbiol 6: 9. 16469109 10.1186/1471-2180-6-9 PMC1513380

[14] DelVecchio VG, Kapatral V, Redkar RJ, Patra G, Mujer C, et al (2002) The genome of the facultative intracellular pathogen *Brucella melitensis* . Proc Natl Acad Sci USA 99: 443–448. 11756688 10.1073/pnas.221575398 PMC117579

[15] Paulsen IT, Seshadri R, Nelson KE, Eisen JA, Heidelberg JF, et al (2002) The *Brucella suis* genome reveals fundamental similarities between animal and plant pathogens and symbionts. Proc Natl Acad Sci USA 99: 13148–13153. 12271122 10.1073/pnas.192319099 PMC130601

[16] Halling SM, Peterson-Burch BD, Bricker BJ, Zuerner RL, Qing Z, et al (2005) Completion of the genome sequence of *Brucella abortus* and comparison to the highly similar genomes of *Brucella melitensis* and *Brucella suis* . J Bacteriol 187: 2715–2726. 15805518 10.1128/JB.187.8.2715-2726.2005 PMC1070361

[17] Denoeud F, Vergnaud G (2004) Identification of polymorphic tandem repeats by direct comparison of genome sequence from different bacterial strains: a web-based resource. BMC Bioinformatics 5: 4. 14715089 10.1186/1471-2105-5-4 PMC331396

[18] Baptista F, Nunes T (2007) Spatial analysis of cattle movement patterns in Portugal. Veterinaria Italiana 43: 611–619. 20422540

[19] Alvarez J, Sáez JL, García N, Serrat C, Pérez-Sancho M, et al (2010) Management of an outbreak of brucellosis due to B. melitensis in dairy cattle in Spain. Res Vet Sci 2: 208–11. 10.1016/j.rvsc.2010.05.028 20579679

[20] Valdezate S, Navarro A, Villalón P, Carrasco G, Saéz-Nieto JÁ (2010) Epidemiological and phylogenetic analysis of Spanish human Brucella melitensis strains by multiple-locus variable-number tandem-repeat typing, hypervariable octameric oligonucleotide fingerprinting, and rpoB typing. J Clin Microbiol 8: 2734–40. 10.1128/JCM.00533-10 PMC2916618 20554816

[21] Aftab H, Dargis R, Christensen JJ, Le Flèche P, Kemp M (2011) Imported brucellosis in Denmark: molecular identification and multiple-locus variable number tandem repeat analysis (MLVA) genotyping of the bacteria. Scand J Infect Dis 43: 536–8. 21375425 10.3109/00365548.2011.562531

[22] Kiliç S, Ivanov IN, Durmaz R, Bayraktar MR, Ayaslioglu E (2011) Multiple-locus variable-number tandem-repeat analysis genotyping of human Brucella isolates from Turkey. J Clin Microbiol 49: 3276–83. 21795514 10.1128/JCM.02538-10 PMC3165627

[23] Whatmore A, Perrett LL, MacMillan A (2007) Characterization of the genetic diversity of *Brucella* by multilocus sequencing. BMC Microbiol 7: 34. 17448232 10.1186/1471-2180-7-34 PMC1877810

[24] Lindstedt BA (2005) Multiple-locus variable number tandem repeats analysis for genetic fingerprinting of pathogenic bacteria. Electrophoresis 26: 2567–2582. 15937984 10.1002/elps.200500096

[25] van Belkum A, Tassios PT, Dijkshoorn L, Haeggman S, Cookson B, et al (2001) Guidelines for the validation and application of typing methods for use in bacterial epidemiology. Clin Microbiol Infect 13: 1–46. 10.1111/j.1469-0691.2007.01786.x 17716294

[26] Clinical and Laboratory Standards Institute (2009) Performance Standards for Antimicrobial Susceptibility Testing: Nineteenth Informational Supplement M100-S19. CLSI, Wayne, PA 19087 USA.

[27] Whatmore AM, Shankster SJ, Perrett LL, Murphy TJ, Brew SD, et al (2006) Identification and characterization of variable-number tandem-repeat markers for typing of *Brucella* spp. J Clin Microbiol 44: 1982–1993. 16757588 10.1128/JCM.02039-05 PMC1489437

[28] García-Yoldi D, Le Fleche P, Marín CM, De Miguel MJ, Muñoz PM, et al (2007) Assessment of genetic stability of *Brucella melitensis* Rev 1 vaccine strain by multiple-locus variable-number tandem-repeat analysis. Vaccine 12: 2858–2862. 10.1016/j.vaccine.2006.09.063 17050051

[29] Martins H, Garin-Bastuji B, Lima F, Flor L, Pina Fonseca A, et al (2009) Eradication of bovine brucellosis in the Azores, Portugal - Outcome of a 5-year programme (2002–2007) based on test-and-slaughter and RB51 vaccination. Preventive Veterinary Medicine 90: 80–89. 19439382 10.1016/j.prevetmed.2009.04.002

[30] Alton GC, Jones LM, Angus RD, Verger JM (1988) Techniques for the Brucellosis Laboratory. Institut National de la Recherche Agronomique (INRA), Paris, France.

[31] Hunter PR, Gaston MA (1988) Numerical index of the discriminatory ability of typing systems: an application of Simpson's index of diversity. J Clin Microbiol 26: 2465–2466. 3069867 10.1128/jcm.26.11.2465-2466.1988 PMC266921

